# An Essay on the Operation of Poisonous Agents on the Living Body

**Published:** 1829-07-01

**Authors:** 


**AN ESSAY ON THE OPERATION OF POISONOUS AGENTS UPON THE LIVING BODY. By *Thomas Addison*, M. D. Assistant Physician to Guy's Hospital, and *John Morgan*, F.L.S. Surgeon to Guy's Hospital. Longman and Co. 1829. Octavo, pp. 91.**

THE Essay which we are about to notice is on a subject which has occupied the attention of the first physiologists of modern times, and more especially since the middle of the last century, when it really became a topic of philosophical enquiry. The investigations concerning the action of poisons on the human body, which were extremely imperfect during the preceding ages, were at length systematically arranged, when toxicology became a science about the period to which we have alluded. It is chiefly indebted to the labours of Fontana, Foderé, Brodie, Orfila, Magendie, and Barry. Few of the medical sciences have been founded so purely on the basis of observation and experiment, and in none have hypothesis and fact been kept more apart, and yet in none have the conclusions been more contradictory and discrepant. We can by no means agree with those who assert, that toxicology has arrived at the dignity of a perfect science; for, in truth, there is much room for further improvement. Similar to all other branches of medical science, this under consideration is famed for the instability of its principles; and even now it is distrusted in many particulars. This assertion will appear much more evident in our subsequent remarks; and we may safely maintain, that the mind of the medical public is well prepared to receive new facts and arguments on this most important branch of medical science. Previous to our offering any remarks on the production before us, we shall briefly remind our readers of the received opinions as to the action of poisons on the system. It is admitted by the most eminent national and foreign physiologists, that poisons act on the human body by one of three ways; 1st, by absorption through the lymphatics; 2d. by venous absorption and by the contact of the deleterious matter with the brain; 3d, by the action of the poison on the sentient extremities of the nerves to which it may be applied, and by their direct communication to the brain. Mr. Brodie, after many scientific experiments, arrived at the second and third conclusions; M. Magendie is the advocate for the circulation of the poison through the blood-vessels; and Dr. Barry agrees in the last opinion, but contends the poison must come in absolute contact with the brain before it can produce its effects. The authors of the work under notice have examined most rigorously the experiments and deductions of the preceding experimenters, and have undertaken to prove their conclusions objectionable and erroneous. They admit the accuracy of the experiments, but maintain that opposite deductions may be drawn from them. They have not rested here, but instituted numerous other experiments which appear to them much more satisfactory in deciding the question at issue. We have perused, with great care and attention, their observations and arguments, and admit they seem to throw strong doubts on the conclusions of their predecessors; but we can by no means give our entire assent to their conclusion, namely, "that all poisons act on the system through the medium of nervous influence only." We freely acknowledge that the present authors have thrown additional light on the obscure subject of their inquiry; but we are decidedly of opinion, that much research is still necessary to convert their theory into a positive certainty. In order to render all parties impartial justice, and more especially to guard the profession from too ready an adoption of any theory, we must submit a full exposition of the objections and conclusions of our authors, and shall accompany them with a few remarks as we proceed.

We need scarcely observe that the inquiry on the action of poisons is of the greatest importance to the medical practitioner, as it embraces the consideration of some of the most obscure and unaccountable phenomena of the animal economy—not only the action of the animal, vegetable, mineral, and aerial poisons, but also the mysterious and inexplicable effects of contagions, and perhaps of the causes of all the diseases incidental to humanity, and perhaps their treatment. Having taken this view of the subject, we shall hope to obtain the indulgence of our readers, for the protracted notice we are induced to take of the work.

The Essay before us is divided into two parts: 1st. On the operation of poisons upon the living body, and a review of the opinions of former writers on the subject. 2d. On the opinions and confirmatory experiments of the authors upon the subject. They have deemed right to explain their reasons for this publication; and we give them in their own words.

"The experiments detailed in this brief Essay were undertaken without any intention of publication. The subject they were intended to elucidate coming within the scope of their duties as public Teachers at Guy's Hospital, the Authors could not but attach to it a lively interest, and were induced to institute the following enquiry, with a view to promote the instruction of their respective Pupils.

"The results, however, of their investigations, have otherwise determined them; for they discover, or imagine they discover, in those results, that which is well worthy the attention of the medical philosopher; and which, they are willing to hope, is calculated to throw additional light upon a subject which must ever deeply concern the physiological, the pathological, and therapeutical Students." Pref. vi.

They next allude to the public clamour against experiments on the lower animals, and apologize very amply, and in our opinion, very unnecessarily to the public, for those which they have instituted. It would almost appear that they meant the apology for the profession, which we need scarcely observe is a work of supererogation. Thus,—

"They with sincerity declare, that nothing but an ardent desire to advance the cause of their profession, and a well-founded hope of its ultimately tending to diminish the sum of human suffering, could have induced them to institute a mode of investigation so painful to every man of correct feeling." vi.

"They, however, are not ashamed to avow, that feelings of humanity have prompted them to economise suffering by varying experiments tending to establish the same position, instead of practising a frequent repetition of such as appeared to them reasonably conclusive in themselves. Neither have they, for similar reasons, deemed it necessary to confirm by experiment what seemed to them satisfactorily established by others. Mr. Brodie has shown, that tying or dividing the lymphatic trunks does not interfere with the operation of poisons on the system; the Authors, therefore, have not considered it expedient to repeat the experiments of that distinguished physiologist, but have confined themselves to the question, whether the absorption of a poison and its subsequent application to the brain are essential to the production of its specific effects upon the body. In conclusion, it may be observed, that if prejudice or preconceived opinion can invalidate testimony, it may probably enhance, in some measure, the confidence of the reader to be told, that, at the commencement of these experiments, the Authors entertained diametrically opposite opinions respecting the question at issue." viii.

Our authors commence their observations by explaining and defining what is a poison, which they conclude in these words:—

"Every substance, then, whether solid, fluid, or aerial, which produces morbid action in the system, is, strictly speaking, a poison: the term, however, is commonly applied to those only of the most active kind." 3.

Few persons will agree to this sweeping and indefinite definition, which embraces all the natural productions subservient to the uses of man, for it is obvious that the most nutritious and innoxious aliments may produce in some habits morbid action, or disorder of function, and yet who will class them among the poisons? We are informed "that the immediate impression made by poisons on the system is a matter which continues to be disputed and obscure," p. 3; but we find our authors, towards the conclusion of their work, very dogmatically and confidently deciding this action on the system.

"The conclusion at which we have arrived is simply this:—

"'*That all poisonous agents produce their specific effects upon the brain and general system through the sentient extremities of nerves, and through the sentient extremities of nerves only; and that when introduced into the current of the circulation in any way, their effects result from the impression made upon the sensible structure of the blood-vessels, and not from their direct application to the brain itself.*'" 60.

But to pursue the remarks of our authors in the regular form, we find an account of the various opinions as to the action of poisons on the system, which may be worthy of insertion for the benefit of our younger readers.

"Now we know that before any sensible effect can be made upon the general system by the application of a local agent, it is essentially necessary that an impression should be carried to the brain by some intervening medium. It has, therefore, become a point at issue amongst former, as well as modern, physiologists, whether the actual contact of the poison with the brain is necessary for its operation, or whether sympathy between the nerves of the poisoned part and the brain is sufficient to establish a communication through which that organ may become affected.

"To bring a poison into actual contact with the brain, it is necessary that the substance should be carried to that organ from the poisoned part through the circulation, either by the medium of the veins, or by the absorbent vessels;—that it should either be conveyed indirectly into the circulating blood, by entering and passing through the absorbents to the subclavian veins; or that it should, on the other hand, directly enter the veins of the poisoned surfaces, and thus be carried immediately into the circulation by passing through those veins into the heart; in which latter case, the poison is said to influence the brain by the medium of venous absorption.

"The advocates for the theory of venous absorption, therefore, suppose that every substance which produces a specific action upon the system, must necessarily enter the veins of the poisoned part, either by what they call an imbibition or soaking through of their coats, or by the capillary suctions of their extreme branches; that having thus fairly penetrated the venous tube, the poison is conveyed in the usual course of the circulation to the heart, and thence to the lungs, where, the blood having undergone a chemical change in other respects, returns, still impregnated with poison, to the centre of circulation, the heart, from the cavity of which organ it is sent by the arteries to the brain; and then, but not till then, does the poison meet with a part of the living solids upon which it is capable of producing a specific influence; and that, therefore, actual contact with the brain by the medium of venous absorption is essentially necessary for the production of those consequences which result from the application of poisons to any part of the living body.

"On the other hand, those who support the theory of nervous communication between the poisoned part and the brain, assert, that the constitutional disturbance arising from the application of a local agent, is adequately accounted for by the connexion or sympathy naturally existing between the extremities of the injured or poisoned nerves, and the sensorium.

"This is, perhaps, one of the most important questions to which a physiologist can direct his attention; it is not confined to the phenomena produced by the action of poisons,—to the operation of arsenic upon the stomach,—to the effects of opium upon the brain, nor to the deadly consequences which result from the wounds inflicted by venomous reptiles; but it involves a theory which has reference to every morbid action that takes place in a living animal, from the operation of local irritation upon the functions of the brain and nervous system: for whether constitutional disturbance shall be produced by the imperceptible operation of noxious miasmata, or whether it shall arise from a visible and local cause, as in the inoculation of small-pox, syphilis, or hydrophobia, still we find no distinct line of demarcation separating the essential characters of what is strictly called a poison, from those produced by more general and more ordinary causes of disease." 8.

We have next a lucid account of the effects of several poisons, as hydrocyanic acid, contagion of plague, and hydrophobia; and of the interval that may elapse between the application of the cause and the development of the effect. In elucidation of this point, we are informed that the most perfect resemblance exists between the symptoms of tetanus, and those caused by nux vomica; between the effects of gun-shot wounds, the contagion of fever, and "the *poisoned* wound of a *venomous* reptile." Thus a slight contused wound inflicted on the bravest soldier will cause such prostration of the vital powers as to extinguish life, and this by the depression of the nervous system through means of its sympathy with the nerves of the injured part. The contagion of plague has acted as fatally, and the bite of a rattle-snake has produced sudden death in the same manner. These facts having been premised, our authors proceed to question the conclusion, that a poison or any ordinary cause of disease shall at one time produce constitutional disturbance through one system of organs, and at another time through the medium of another. This is the question at issue, and therefore the authors shall speak for themselves.

"Analogy, therefore, as regards *effect*, can be perfectly established between the consequences of a local injury, those which are produced by the poisoned wound of the more venomous reptiles, and those which arise from the influence of an ordinary cause of disease. Therefore the analogy between poisons and other agents capable of exciting morbid action in the system is complete, as well as it regards their sensible effects, as in reference to the interval of time which may elapse between the application of their causes, and the development of their effects." 14.

"All fair analogy forbids the conclusion, that a poison or an ordinary cause of disease shall at one time produce constitutional disturbance through the medium of one system of organs, and at another time through the medium of another system of organs: that, under certain circumstances, a poison or any other cause of disease shall affect the functions of the brain and nervous system, at one time through the medium of the nerves, at another time by the circulation, and at another by the action of the absorbent vessels: that at one time a poison shall be taken up by the veins, and carried through the circulation to the brain, before it produces any sensible effect: that at another time the absorbent vessels shall take up the substance, and, by their communication with the subclavian veins, be thus instrumental in carrying the specific agent into the circulation, and thence to the brain: and that again at another time the impression made upon the extremities of the nerves of the poisoned part shall at once, by the medium of those poisoned nerves, be conveyed to the brain, independently of absorption either by the veins or absorbent vessels. It is contrary to all fair analogy to suppose that any variety observed in the *effect* of a local agent can essentially depend upon the medium by which it is carried into the system.

"As reasonably might it be presumed that at one time the sense of taste was communicated by a branch of the fifth pair of nerves, and at another time by the salivary ducts, as to entertain a belief that veins, absorbents, and nerves individually performed a function of precisely a similar nature.

"On the contrary, every organ in the living system is destined to perform its proper functions, and, as far as our physiological researches have yet extended, we are justified in assuming that no two organs in the human body are capable of performing the same function, neither can two functions be performed by the same organ.

"It will not, for instance, be said that the sympathy between the brain and stomach is conveyed along the eighth pair of nerves at one time, and by the circulating system at another: that when vomiting occurs as a consequence of concussion of the brain, it is occasioned by nervous sympathy; and that when morbid sensibility of the brain is produced by derangement in the digestive organs, that morbid sympathy is the result of absorption: that there are two roads between these two organs, by which sympathy is established; the one leading from the head to the stomach, and the other from the stomach to the head.

"Since, then, the various phenomena resulting from the effects of poisons upon the system are too intimately connected with those produced by other causes of morbid excitement to admit of any distinct line of separation; since we are led by analogy to believe, that the medium by which an impression is made upon the brain and nervous system is the same in both cases, it must be manifest that the question, which has arisen amongst physiologists in every age, respecting the medium through which poisons influence the general system, is one that has reference also to the mode in which all morbid phenomena are produced in the living system; and, therefore, it involves a theory of the highest importance to the physiologist, to the physician, and to the surgeon; a theory, indeed, intimately and inseparably connected with every branch of science which has for its object the elucidation of those phenomena produced by local agents of every description upon the living body." 18.

The opinions of the earlier physiologists, Fontana, Orfila, and others, are allowed to have afforded much valuable information on the effects of the different poisons; but as to the medium through which those effects are produced, they have left the inquiry where they found it, involved in doubt and obscurity. The received opinions are again detailed, and in conclusion we are informed, "that popular opinion and modern publications favour the theory of venous absorption." We cannot readily comprehend what degree of weight is due to popular opinion, on the intricate subject before us, as we have received an early impression, that public fame is worthy of little credence on any occasion, and of none in philosophical discussion. Our authors next proceed to analyse the experiments and opinions of Mr. Brodie, M. Magendie, and Dr. Barry, and certainly with much ability and judgment, but we must decline offering an opinion in support or defence of the parties at issue, all of whom are so well able to defend themselves, and shield ourselves under the adage of a classic writer,—

Non nostrum inter vos tantas componere lites.

To proceed then to the subject:—we have to inform our readers, that some of the experiments of Mr. Brodie are first examined, especially those on which he has formed his opinion. We cannot avoid inserting them as detailed in the work before us.

"In the first experiment, the essential oil of bitter almonds was applied to the tongue of a cat, in consequence of which the life of the animal was instantly destroyed.

"The second experiment consisted in the injection of the same poison into the rectum of another animal of the same species, by which death was occasioned in two minutes.

"In neither case were any morbid appearances found on dissection.

"From these experiments a conclusion has been drawn, that the poison acted upon the brain by the medium of the nerves." 21.

To the deductions made by Mr. B. our authors object in no gentle terms.

"But we must protest against the train of reasoning by which the theory of nervous communication has, in this instance, been supported; for in support of that theory it is assumed, that the susceptibility of any part of the human body to a morbid impression may be correctly estimated by a dissection of its nerves, and that in proportion to the greater size and number of those nerves will be the rapidity with which such morbid impression is conveyed to the brain. We must also dispute the assumption, that the extreme irritability of the nerves of the tongue, under one proper cause of excitement, is to be considered as a proof of their increased excitability to action under the impression of a morbid agent.

"Each assumption appears to us to be equally untenable, and to involve a theory respecting the multiplied functions of a single nerve, which is not only unsupported by analogical reasoning, but which is in many instances directly contradicted by fact.

"Analogy teaches us, that to each fibril of a nerve a separate office is assigned; for we have not a single instance to prove that any one nervous filament in the living system is capable of essentially performing a double function." 23.

To controvert the conclusion of Mr. B. the following experiment was instituted.

"The spinal marrow of a half-grown rabbit was divided; the leg was inoculated with strong Prussic acid: the animal died in three minutes after the introduction of the poison, this being the usual period of time in which that poison was found to operate upon these animals under common circumstances when introduced into the same part.

"Now, if the impression produced upon the brain by the application of

Prussic acid to a distant part be the consequence of its action upon nerves of sensation and voluntary motion, as has been supposed to be the case by Mr. Brodie in his experiment, we should be happy to receive from the advocates of that gentleman's theory a satisfactory explanation of the result of the experiment just related.

"If, on the other hand, the impression of the poison be carried to the brain by other organs, we, for the present, leave the advocates of venous absorption and infiltration to account for its instantaneous effect upon the system when applied to the mouth." 26.

Mr. Brodie was of opinion, that a poison may act on the brain by entering the circulation through the divided veins of a wounded surface. Having tied the thoracic duct of a rabbit, he inoculated its leg with ticunas, and the poison proved fatal in the usual time; and he therefore concluded the action could not have been through the absorbent vessels. He also maintains that a poison must enter the substance of the brain through the circulation of the blood. In proof of this opinion, he tied a tape half an inch wide round the thigh of a rabbit, having excluded the sciatiatic nerve; the leg was then poisoned with ticunas; no sensible effect was produced at the end of half an hour, the ligature was then removed, and in twenty minutes the animal was found motionless and insensible. To disprove this opinion the following experiment was made.

"Four drops of Prussic acid were applied to the wounded foot of a full-grown rabbit, by which death was produced in two minutes and a half.

"In another rabbit of the same age a ligature was tied round the leg, to the careful exclusion of the sciatic nerve; six drops of the same poison were then applied to a wound in the foot of the strangulated limb, and without producing the slightest sensible effect.

"Mr. Brodie is satisfied that the poison of Prussic acid acts upon the brain by the medium of nervous communication; it is manifest, however, from the foregoing experiment, that, according to the view which he has taken of the subject, the same argument by which the theory of venous absorption is supported in reference to the poison of woorara, holds equally good in this case as applied to the poison of Prussic acid.

"Either, therefore (according to Mr. Brodie's train of reasoning), we have a direct proof that Prussic acid does not act upon the brain by the medium of the nerves, or we must consider his theory respecting the venous absorption of other poisons to be entirely unsupported by the result of the experiment upon which that theory was principally founded; for, according to the position which Mr. Brodie has taken, the application of Prussic acid to the strangulated limb ought to have produced its usual effect upon the system; whilst the division of the spinal marrow, according to his views of the subject, ought to have precluded the possibility of any nervous communication between the brain and any part of the lower extremity to which that poison had been applied; yet, in both cases, we find that such a supposition is directly opposed to the evidence of fact.

"Whether, therefore, the poison of woorara affects the system through the medium of nerves, or whether it be carried to the brain by the circulation, is a question which cannot be satisfactorily settled by the experiment which Mr. Brodie has instituted, for the purpose of settling that disputed point, since the explanation he has given of their results is directly contradicted by the other experiments mentioned." 33.

Mr. Brodie's theory of the absorption of a poison through a divided vein is also opposed, and decided to be "physically impossible." We must here insert the reasons assigned in the words of the authors, as we find it impossible to condense, or rather to comprehend the ambiguity of the two following sentences. We are not disposed to criticise severely the style of modern writers, but we cannot but animadvert on the extraordinary length of most of the sentences of the present work, which often renders the meaning obscure, and confuses the mind with the multiplicity of opposite facts and opinions which are included in a single period. We offer the subsequent examples in justification of this remark.

"When a vein is divided, it is well known, that unless some branch be interposed between the truncated extremity and the next valve in its course towards the heart, unless the current of blood be driven through the cut extremity of the tube by collateral branches, we find that the sides of the vessel as far as the next valvular interruption will collapse and remain inactive: supposing the poison, then, to enter this flaccid tube, it is completely prevented from mixing with the circulating blood which fills the vessel above the valve by the pressure made by that circulating blood upon the opposite side of the valve, and, consequently, under such circumstances, unless it can be proved that a poison has the property of propelling itself, it requires no argument to prove that the substance, instead of passing into the circulation, will remain stationary in that part of the vessel through which no circulation is carried on. If, on the other hand, circulation be carried on by collateral branches through that part of the tube which lies between the nearest valve and the mouth of the divided vein, the effect will be still more unfavourable to Mr. Brodie's hypothesis; for the circulation in that part of the vessel will be reversed, and, consequently, instead of being carried towards the heart, the poison will be washed out of the wound: in either case, therefore, it seems highly improbable that the poison can pass through the circulation to the brain by the medium of a divided vein." 34.

Again we find it impossible to condense the concluding censure on Mr. B.

"With regard to the general theory of venous absorption, as supported by Mr. Brodie's experiment of applying a ligature to a limb which has been poisoned, we have to observe, that the experiment will admit of a different explanation from that with which he has furnished us; for since it is proved that the nerves of sensation and volition are not necessarily concerned in carrying to the brain the impression of a certain poison, which he admits must affect the system by nervous communication with the sensorium; since it is therefore obvious that other nerves, performing different functions, must be concerned in the operation of that poison upon the brain; since we have proved that a ligature placed around the limb, to the exclusion of the sciatic nerve, in the manner above stated, produces the double effect of paralysing the nerves upon which one poison operates, as well as of stopping the circulation through the part, it may be fairly inferred, that if the other poison should happen to act upon the brain by the medium of the same set of nerves, the operation of that poison during the period of strangulation would be suspended from a similar cause, and that, consequently, this experiment relative to the absorption of woorara is quite inconclusive as supporting the truth of either theory." 36.

Having disposed of Mr. B.'s opinions, our authors proceed to examine those of M. Magendie, the substance of which is, that the admixture of all poisons with the blood in the poisoned part is absolutely necessary to their operation on the body; and this deduction was made from the following experiment, which our authors "oppose in the strongest possible manner." A dog was stupefied with opium, and the femoral artery and vein were divided, but re-united by pieces of quill which were secured in the truncated vessels, the limb was amputated, all nervous connexion with the body of course destroyed, but the circulation was uninterrupted. The poison of upas was applied to a wound in the severed limb, and produced its deleterious effects in the usual time. Pressure was made occasionally on the vein, when the symptoms of poisoning ceased, but returned after the removal of the pressure. This experiment appeared perfectly conclusive as to the absolute necessity of venous absorption, as the medium of conveying the action of the poison to the brain. The substance of our authors' objection to this experiment is, that if a dog be stupefied by opium, it is impossible to determine, if upas be applied, whether the animal shall have been destroyed by the one or the other, or by the combined action of the two. Again it is maintained, that the poison must have acted on the inner surface of the veins and arteries before it could reach the brain; or, in other words, on the capillaries or nerves of such surface. It is also doubtful, whether the brain, or the nerves of the vein to which the poison has been applied, receive the first impression. We hope we have been correct in stating the meaning of the passages, which we have condensed—on the whole, the experiment only proves the entrance of the poison into the vein; nor is it to be inferred, that such application of a poison is absolutely necessary to its operation. Our authors conclude in these words:—

"We contend, then, that M. Magendie has left the question relative to the necessity for venous absorption and cerebral contact, as connected with the operation of poisonous agents, in precisely the same state as he found it." 50.

The opinions of Dr. Barry are next noticed, and the following experiment commented on. Dr. B. introduced four grains of upas tieuté into a wound on the thigh of a small dog; and applied a cupping-glass and piston to a similar wound, but unpoisoned, in the opposite thigh. Symptoms of poisoning took place in eight minutes. The cupping-glass was then removed to the poisoned wound, a vacuum established, and instantly the symptoms were alleviated. "The animal was truly recalled to life, but from time to time suffered slight attacks of tetanus. After a quarter of an hour the glass was removed, and the animal appeared restored to health." Our authors observe in italics, "the animal was found dead some hours afterwards;" and they cannot comprehend the reason of applying the cupping-glass to the simple wound in the unpoisoned limb. Dr. Barry concluded from this and many other experiments, that the circulation through the extreme branches of veins is kept up by atmospherical pressure upon the surface of the body, and that venous absorption is necessary as a means of communication between a poisoned surface and the brain. To the last conclusion our authors object in these words:—

"It will, we think, be admitted by every one, that the soft structures of the surface of the body, which are covered by an exhausted cupping-glass, must necessarily, from the pressure of the edges of that glass, be deprived for a time of all connection, either nervous or vascular, with the surrounding parts. That the nerves must be partially or altogether paralysed by compression of their trunks, and that, from the same cause, all circulation through the veins and arteries situated within the area of the glass must cease; this, however, is not the only change which is produced in a part by the mode in which Dr. Barry has, in his experiments, removed from it the pressure of the atmosphere; for, not contented with merely stopping the circulation, we are informed that the rarification of the air within the glass was still further increased by means of a small air-pump attached to it, so that the fluids contained in the divided extremities of the vessels were forced into the vacuum, and with these fluids, of course, either a part or the whole of the poison which had been introduced.

"In such a condition of parts, it will be manifest that the compression on the one hand, and the removal of the poison from the wound on the other, will explain in a very satisfactory manner the result of the experiment, as well to the advocate for nervous communication, as to the supporter of the theory of venous absorption. For if the extreme branches of the nerves of a wounded part be paralysed by the pressure of a cupping-glass, of course no sympathy can be established between those nerves and the brain. Or, if the poison be entirely removed from its contact with the nerves by the formation of a vacuum over the wound, we may reasonably suppose that the cause of irritation being no longer in operation, the effect will be no longer apparent; and, consequently, whether the impression made upon the system be the effect of the actual contact of that poison with the brain by the medium of venous absorption, or whether it be produced by nervous sympathy, we may in either case expect from Dr. Barry's experiments precisely the same result as he has described to us." 57.

There is a good deal of force in the explanation offered by our authors on the effect of the cupping-glass in the prevention of the action of poisons; but we cannot admit that the confirmation of that fact, or rather the revival of the remedy, is not something novel in the treatment of poisoned wounds —no matter how the action of the poison is prevented, whether it be by the cessation of the functions of the lymphatics, veins, or nerves of the injured part; the knowledge of the fact is worth all the theories that have ever been offered on the subject. If the remedy prove equally efficacious in hydrophobia as it has in the numerous instances in which animals have been poisoned by venomous reptiles, and of this there is the strongest probability, we cannot help thinking, that Dr. Barry has added something new, and added more real and solid practical information on the treatment of poisoned wounds, than all the writers on toxicology collectively. We by no means assent to all that gentleman's theories; but we fully agree with our Northern contemporary that "he is entitled to a very high rank among the ablest physiologists of the present day."* With respect to the opinions of Mayo and others it is said they contain nothing but the re-statement of those just adverted to, and therefore it is unnecessary to notice them separately; and thus conclude the preliminary observations on the opinions of former toxicological writers. We now arrive at the second part of the work, which is on the opinions of the authors upon the subject of poisons. Here we deem it right to insert their own language.

"Hitherto we have refrained from declaring any decided opinion whatever as to the *modus operandi* of poisonous agents on the living body; but having assumed it to be unphilosophical to admit a two-fold operation—to admit that such agents may at one time act upon the general system, through the medium of the sentient extremities of nerves, and at another time by the direct application of the poison to the brain, through the medium of the blood; and having attempted to show the fallacy of those experiments upon which the notion of a two-fold operation of poisons has been founded,—we must now venture to acknowledge an attempt to establish the truth of a theory, which we conceive to be supported as well by the experiments of our predecessors, as by those which we have ourselves instituted upon the lower classes of living animals." 60.

* Edinburgh Medical and Surgical Journal, 1827, v. 27.

"In endeavouring to establish the truth of this our theory respecting the *modus operandi* of *all* poisonous agents, we cannot be insensible to the powerful array of authority which is opposed to us, nor to the influence which that authority has probably exerted upon the minds of all our readers; we feel, therefore, that we have to contend with the prejudices as well as with the arguments of our opponents.

"Brodie, Magendie, and Barry all appear to be quite satisfied, that in many instances the venous absorption and direct application of a poison to the brain are absolutely necessary to its operation upon the general system; although they are compelled to admit that some poisons act at once through the sentient extremities of nerves. Dr. Barry, however, has particularly laboured to show in how short a time a poison may reach the heart through the medium of venous absorption; a circumstance calculated, if not really intended, to cast a shade of doubt upon the operation of poisons through the nerves in any case." 61.

We have already given the theory of Dr. Addison and Mr. Morgan, namely, that, "all poisons act on the system through the medium of nerves only." They are surprised that former physiologists should not have admitted this theory. They had seen it exemplified in the effect of opium on a pained part, of belladonna on the iris, and in certain phenomena which take place in disease. Thus—

"A person receives a slight lacerated wound, a burn, a puncture from a spicula of wood, or a rusty nail; the irritating cause being removed from the part, all shall appear to be going on well, when suddenly symptoms of tetanus supervene, and proceed to the destruction of life: here we have, then, the mere irritation of the nerves of a small portion of the body, so deranging and involving the entire nervous system, as to give rise to one of the most formidable of all diseases, and this, so far as we know, without the slightest evidence of any absorption of morbid matter into the current of the circulation, and with just as little evidence of any thing noxious being directly applied to the brain. If, then, mere irritation, mechanical or otherwise, prove sufficient to derange the whole nervous system in this instance, where, we would ask, is the difficulty of conceiving that morbid irritation, and consequent general derangement, in the system, should result from the application of a poison, altogether independently of any absorption whatever?" 65.

These are strong facts in support of the proposed theory. Again we are reminded how many poisons act too suddenly for the medium of absorption. It is said, that the exclusion of the sciatic nerve in a limb included in a tight ligature as already described, rendered that nerve incapable of performing its functions in the absence of all circulation. This is a strong objection, but one that equally applies to the experiments of our authors. Of this hereafter.

In confirmation of our authors' deductions the following experiments were instituted.

"To prove the extreme susceptibility of the inner coat of a vein, when exposed to the action of a poison, the following experiments were made:—

"The jugular vein of a full-grown dog (in size about that of a common harrier) was laid bare to the extent of about two inches; the circulation through the denuded vessel was then completely stopped by the application of two temporary ligatures, one of which was tied round the upper, and the other round the lower, part of the exposed vein; the vessel was then divided between the two ligatures, and the truncated extremities reconnected by means of a short brass cylinder or tube, within which was placed a portion of woorara, of the size of a grain of canary seed. In this way the continuity of the canal between the temporary ligatures was preserved entire, the brass tube being inserted and tied within the mouths of the cut extremities, and, consequently, allowing, on the previous impediment to circulation being removed, a free passage of blood from the upper to the lower part of the vein; with which blood, of course, the poison in the interior of the tube would instantly be mixed, and carried through the circulation.

"Both the temporary ligatures being then removed, the accustomed circulation through the vessel was re-established; and in forty-five seconds the animal dropped on the ground, completely deprived of all power over the muscles of voluntary motion; in two minutes convulsions and respiration had entirely ceased.

"We were perfectly aware, in making this experiment, that the result might be adduced in proof of the truth of the theory of the cerebral contact, as well as of nervous communication of a poison; it was, therefore, merely made for the purpose of affording a contrast to the following:—

"The jugular vein of a dog, of the same size and age with the preceding (both being from the same litter), was exposed, and separated from its surrounding connection, to the extent of three inches; temporary ligatures were then applied, as in the former case. A small opening was then made in the vein, immediately above the lower ligature, through which a cylinder of quill was pushed into the interior of the vessel; within this quill a piece of woorara, of the same size as that used before, had been previously inserted. The vein was now again tied by a permanent ligature above the opening, through which the quill had been thrust, leaving a space of two inches and three quarters between the first upper temporary ligature and the permanent one last mentioned; the interior of the vessel between the two containing the poison which was thus prevented from coming in contact with the sides of the vessel, until washed by blood out of the quill in which it was contained. The upper temporary ligature was next removed, so as to allow the blood to pass down into the lower part of the vessel as far as the lower permanent ligature, and, consequently, to that part of the vein which contained the poison.

"Now it must be manifest, that the solution of the poison in the blood, under such circumstances, could only act upon the system through the vessels or nerves of the *vein;* for the direct entrance of the poisoned blood into the heart, &c. was prevented by the lower ligature, and we, consequently, ought to have found, in the case of venous absorption, the effect nearly the same as that which would have occurred from the introduction of the poison to any other part of the body, in which a capillary absorption, and, consequently, a greater length of time, was necessary to its operation; for it will be remembered, that circulation was no longer going on through the trunk of the jugular vein itself.

"It was found, however, on the contrary, that in the space of 108 seconds after the removal of the ligature the animal dropped in convulsions, as in the former case, and expired in three minutes and a quarter.

"The poison which was used in these two cases has never been known in dogs to produce a sensible effect upon the system, in cases of its insertion into superficial wounds of the body, in less than six minutes, and respiration usually has ceased in from a quarter of an hour to twenty-five minutes.

"Now, if we suppose that the poison of woorara produces its effect upon the system through the medium of the nerves, we can easily reconcile with that supposition the results of our two experiments: for we may attribute, in that case, the rapid operation of the poison in the last experiment, as compared with its effects upon a superficial wound of the body, to its contact with the acutely susceptible extremities of nerves distributed to the inner coat of the jugular vein itself; and we may also account for the *extreme* rapidity of its action in the first experiment, by its contact with the more extended and equally susceptible surfaces to which it must have been necessarily applied in the course of its direct circulation through the vena cava, the heart and arteries." 74.

As the intimate connexion between the nervous and vascular systems offer great difficulties to conclusions drawn from experiments made on either, our authors have endeavoured to obviate this impediment by attempting, as it were, to separate such connexion, and instituted experiments for the purpose.

"Two large bull-dogs, of equal size and strength, were held face to face upon a table, embracing each other; so that their breasts and necks were in contact, the animals being placed upon their sides. In this position, it will be seen that the right carotid of one dog and the left of the other were uppermost, and that these vessels, when exposed by operation, might, while the animals were thus held together, be brought into contact. It was therefore our object to establish a connection and a circulation between these two arteries, viz. between the right carotid of one dog, and the left of the other.

"In this way, then, we established a circulation of blood from the heart of one dog to the head of the other; and it was therefore reasonable to suppose, according to the theory of the supporters of venous absorption and cerebral contact, that the dog contributing blood to the other, would, after inoculation with a poison, be supplying his neighbour with poisoned blood, which reaching the substance of the brain, must necessarily produce the same effects in the one as in the other. This, however, was not the case; for upon introducing the poison of nux vomica into the back of the animal, from whose carotid the blood was passing to the opposite vessel of the other dog, we found that although the usual violent effect was produced in the inoculated animal, and although that effect continued for the space of fourteen minutes, during which period a free circulation was carried on between them in the manner already mentioned, yet that not the slightest indications of the action of the poison upon the system could be observed in the other dog.

"Satisfied that the experiment had been continued long enough, the artery was then tied in the neck of the sound dog, the vessel was divided, and the sufferings of the other and expiring animal were terminated. On the following day the surviving dog continued free from all symptoms of poisoning.

"It can hardly be supposed in this case, that if the poison had been dissolved in the blood which circulated through the carotid of the poisoned dog, no portion of it would have reached the brain of the other, since it was repeatedly seen during the operation that a free current constantly passed through the vessels from one animal to the other; indeed if such had not been the case, it must be manifest that coagula would have formed in them, as well as in the brass tube by which they were connected. In the case, therefore, of sanguineous contamination and cerebral contact, we think it fair to infer that both animals ought to have been affected by the poison in nearly the same way, and at the same time, the opposite of which was proved to be the case.

"This experiment having satisfied us that the circulation of a poison through the brain was not the cause of its operation upon the body, we next proceeded to ascertain, by a nearly similar mode, whether in all cases the contamination of the blood in the veins, which supplied a poisoned part, was necessary to the operation of the poison upon the system.

"Two large hounds were secured neck to neck, as in the former case, and the division and re-connection of the jugular veins effected in a manner precisely similar to that already described in the case of the double circulation through the carotid, so that the venous blood from the head of one dog passed into the heart of the other. The animal contributing blood to the other was then inoculated on the side of the face with nux vomica, and in the usual time exhibited the usual symptoms: these continued without intermission during the space of seven minutes after the animal was first affected, the circulation being freely kept up through the artificially-connected jugulars: at the end of this period the circulation was beginning to become impeded by the formation of a small coagulum in the tube, and we therefore terminated the experiment by destroying the poisoned animal, the other dog never having shown the slightest symptoms of being poisoned. Now, if in this case the poison had been taken by the veins in a sufficient quantity to affect the distant textures to which it was carried in its course through the circulation, we do not think it possible that either dog could have escaped; at all events, if either had escaped, it would have been the poisoned animal, whose veins were prevented carrying contaminated blood from the part through the system by their connection with those of another." 86.

A double circulation was effected by connecting the lower end of the divided carotid artery of one dog, with the upper end of the carotid of another, so that each animal supplied the brain of the other; and consequently the poisoned dog received from the unpoisoned animal a supply of blood equal in quantity to that with which he was parting. The form of the connexion is shewn by a plate, a rude idea of which may be conveyed by the letter X; the division of the arteries being above the superior angle, and the left side of the letter representing the carotid of one dog, the right that of the other. The experiment was as follows:—

"Two large half-bred bull dogs, each weighing about forty pounds, were the animals selected for the operation. The carotid artery of each dog having been laid bare on one side, and separated from its connections with surrounding parts to the extent of three inches, temporary ligatures were applied above and below, and the arteries were divided between them, as in a former case; the brass tubes were then attached to the extremities of the vessels, and the necks of the two animals being held and closely bound together, the divided arteries were, without the least difficulty, re-connected, and the circulation reversed.

"One of the dogs was then inoculated on the back with a concentrated preparation of strychnine, which had been found upon other occasions to produce death in these animals in about three minutes and a half.

"In three minutes and a half the inoculated animal exhibited the usual tetanic symptoms which result from the action of this poison, and died in a little less than four minutes afterwards, viz. about seven minutes from the time at which the poison was inserted, during the whole of which time a free and mutual interchange of blood between the two was clearly indicated by the strong pulsation of the denuded vessels throughout their whole course.

"The arteries were next secured by ligature, and the living was separated from the dead animal; but neither during the operation, nor at any subsequent period,* did the survivor show the slightest symptom of the action of the poison upon the system.

"From these, then, and from many other similar experiments, which it would be needless to instance, we have been led to the conclusion that all poisons, and, perhaps, indeed, all agents influence the brain and general system, through an impression made upon the sentient extremities of the nerves, and not by absorption and direct application to the brain.

"So far as we are competent to judge, the conclusion is borne out by the experiments detailed in this Essay; but if those experiments be not satisfactory to our readers, we at least indulge the hope, that what has been advanced may lead to the discovery of more satisfactory and more conclusive evidence than has yet been adduced on this truly important question; a question, indeed, of the deepest interest, not as a mere matter of curiosity, but as involving the elucidation of many of the most prominent, and at present the most mysterious phenomena of a living body.

* "This animal was killed on the following day."

"If found to be correct, the principle for which we contend will not be limited to the operation of those noxious agents usually denominated poisons, but it may probably tend to the better understanding both of the causes and cure of diseases in general." 91.

Such are the experiments and conclusions of our authors in proof of their theory. We have once more to apologize for devoting so much space to the review of the Essay before us, but the vast importance of the subject, and impartial justice towards the individuals at issue, absolutely required it. We think the objections and arguments employed by Dr. Addison and Mr. Morgan against those who maintain opposite opinions, very powerful, and such as require answers, if any can be given; while their experiments are ingenious and deeply interesting, their deductions in general are natural and legitimate, and their theory on the whole as well, if not better supported, than any other which has been received. We think, however, it is by no means fully established, and that much more research is necessary to entitle it to adoption. It has been contended that a nerve deprived of circulation, as the sciatic in a former experiment, could not perform its functions; but were not the nerves of the isolated jugular veins in our authors' experiments in the same condition, and besides subjected to pressure for a considerable time? According to their own shewing, these nerves could not perform their functions, and it is not easy to admit, that nerves even remote in the same vessels, must not be affected by sympathy, and must not have their functions deranged by the injuries inflicted on the divided vein and surrounding parts during the experiments. The experiments upon the whole seem to us to disprove the doctrine of poisons acting by absorption. But though we think our authors have thrown much light on the action of poisons on the system, we regret that they have left the more important matter, the prevention of such action, as they found it.

